# Predictors of Long-Term Disease Control and Survival for HER2-Positive Advanced Breast Cancer Patients Treated With Pertuzumab, Trastuzumab, and Docetaxel

**DOI:** 10.3389/fonc.2019.00789

**Published:** 2019-08-22

**Authors:** Ashley M. Hopkins, Andrew Rowland, Ross A. McKinnon, Michael J. Sorich

**Affiliations:** College of Medicine and Public Health, Flinders University, Adelaide, SA, Australia

**Keywords:** anti-HER2 therapies, advanced breast cancer, prognostic model, pertuzumab, trastuzumab, survival, progression

## Abstract

**Background:** HER2-positive advanced breast cancer (ABC) is associated with significant heterogeneity in long-term disease control and survival. Prognostic models for HER2-positive ABC patients considering first-line pertuzumab, trastuzumab, and docetaxel have not been evaluated.

**Methods:** A pre-treatment prognostic model for progression-free survival (PFS) and overall survival (OS) was developed for HER2-positive ABC patients initiating first-line pertuzumab, trastuzumab, and docetaxel using clinicopathological data from the randomized clinical trial CLEOPATRA (*n* = 408). Cox proportional hazard analysis with a backwards deletion process was used.

**Results:** Metastatic sites count (<3 vs. ≥ 3) and lactate dehydrogenase (LDH) (≤ ULN vs. >ULN) were identified as common pre-treatment risk predictors for PFS and OS (*P* < 0.05). Based on these two factors, patients can be characterized as one of three prognostic groups (good = 0 factors; intermediate = 1 factor; poor = 2 factors). The prognostic groups were associated with significantly different PFS (*P* < 0.001), with 3-year PFS probabilities of 44% (36–55), 28% (22–36), and 17% (11–29) for the good, intermediate and poor prognostic groups, respectively. Similarly, there was significant differences in OS (*P* < 0.001), with 4-year OS probabilities of 75% (95% CI: 67–84), 60% (53–68) and 31% (21–45) for the good, intermediate and poor prognostic groups, respectively.

**Conclusions:** Pre-treatment prognostic groups identified for HER2-positive ABC patients initiating first-line pertuzumab, trastuzumab, and docetaxel had significantly different long-term disease control and survival outcomes.

## Introduction

Current consensus for the treatment of HER2-positive (human epidermal growth factor receptor 2) advanced breast cancer (ABC) is that anti-HER2 therapy should be offered first-line to all patients, unless contraindicated (1). The choice of specific anti-HER2 agent will depend on country-specific availability, patient and clinician preferences, and previously administered therapies during early disease and responses to them (1). A recommended first-line therapy option for HER2-positive ABC is the combination of pertuzumab, trastuzumab, and docetaxel which was observed to improve progression free survival (PFS) and overall survival (OS) compared to trastuzumab and docetaxel in the CLEOPATRA study (1–4). Although HER2-positive ABC is associated with relatively good prognosis compared to other forms of advanced cancer, there is still substantial heterogeneity between patients with respect to prognosis and benefit from treatment.

Multivariable risk prediction tools integrate information from multiple patient and tumor characteristics to identify patient groups with different prognosis (5). Thereby, clinical risk prediction tools may facilitate improved decision making by providing patients with personalized realistic expectations of long-term disease control (e.g., probability of 3-year PFS) and survival (5–7). Although a number of individual prognostic factors have been associated with therapeutic outcomes in HER2-positive ABC patients (8–11), there are no validated clinical risk prediction tools applicable to HER2-positive ABC patients being considered for first-line pertuzumab, trastuzumab, and docetaxel treatment.

The objectives of this study were to identify pre-treatment HER2-positive ABC patient groups with distinct PFS and OS probabilities when initiating first-line treatment consisting of pertuzumab, trastuzumab, and docetaxel.

## Materials and Methods

### Population

Individual-participant data (IPD) from the Roche sponsored clinical trial CLEOPATRA (NCT00567190) (2–4) were accessed via clinicalstudydatarequest.com. Secondary analysis of anonymized participant-level trial data was approved by the Southern Adelaide Clinical Human Research Ethics Committee.

Development data for the pre-treatment prognostic models consisted of HER2-positive advanced breast cancer patients (*n* = 408) enrolled in CLEOPATRA who initiated intravenous (IV) pertuzumab at 840 mg for cycle 1 followed by 420 mg for subsequent cycles, plus IV trastuzumab at 8 mg/kg for cycle 1 followed by 6 mg/kg for subsequent cycles, plus 75 mg/m^2^ of IV docetaxel for each cycle (cycle length = 21 days) (2–4). Each of pertuzumab, trastuzumab, and docetaxel were continued until investigator-assessed radiographic or clinical progressive disease, or unmanageable toxicity (2–4). If an agent was discontinued for toxicity, treatment with the remaining agents was permitted (2–4).

### Predictors and Outcomes

Analyzed covariates were prespecified based upon availability, prior evidence (8–11) and biological plausibility. Analyzed pre-treatment covariates included age, Eastern Cooperative Oncology Group performance status (ECOG; 0 or ≥1), race, estrogen and progesterone receptor status, visceral disease (no or yes), time since diagnosis, any prior trastuzumab or taxane in all settings, count of metastatic sites, lactate dehydrogenase (LDH), and neutrophil to lymphocyte ratio (NLR).

PFS was defined from the time of randomization to disease progression or death from any cause, with progression assessed by the investigators using the Response Evaluation Criteria in Solid Tumors (RECIST) version 1.0 (4). OS was defined from the time of randomization to the last follow-up or death from any cause (4).

### Statistical Analysis

Univariate Cox proportional hazard analysis was used to assess the association between potential predictors and PFS/OS. Associations were reported as hazard ratios (HR) with 95% confidence intervals (95% CI), and *P*-values (likelihood ratio test). A major objective of the study was to develop an easy to use pre-treatment prognostic model. Therefore, there was a preference to minimize numerical derivation / subgrouping while maintaining predictive performance. Visual checks were used to assess potential non-linear effects of continuous variables, with cut-points selected based upon discriminative performance (concordance statistic) and standard clinical practice stratifications.

A pre-treatment prognostic model was developed using multivariable Cox proportional hazards analysis. Separate prognostic models were initially developed for PFS and OS via a backwards deletion of significant univariates (*P* < 0.05). Coefficients and *P*-values for the optimal prediction models were reported. Subsequently, simplification of the developed PFS and OS models were explored. To facilitate clinical translation (i.e., minimize computation), a single model for PFS and OS, with minimal numerical derivation/subgrouping and maintained predictive performance was to be preferred.

Discriminative performance of defined pre-treatment prognostic models was assessed via the concordance statistic. Kaplan–Meier analysis was used for plotting and estimating probabilities. Data analysis was conducted using R version 3.3.0. (12).

## Results

### Patient Population

Supplementary Table 1 provides a summary of the patient characteristics for the 408 HER2-positive ABC patients treated with first-line pertuzumab, trastuzumab, and docetaxel from CLEOPATRA. Median follow-up was 49 [95% CI: 48–51] months within the cohort.

### Univariate Analysis

Univariate Cox proportional hazard analysis identified prior taxane therapy, metastatic sites count, LDH, and NLR as pre-treatment prognostic markers of PFS in HER2-positive ABC patients commencing first-line pertuzumab, trastuzumab, and docetaxel (*P* < 0.05; Supplementary Table 2). ECOG performance status, ER and PR status, metastatic sites count, and LDH were identified as pre-treatment prognostic markers of OS (*P* < 0.05; Supplementary Table 2).

### Pre-treatment Prognostic Model

The backwards deletion process resulted in a PFS pre-treatment prognostic model which included metastatic sites count (<3 vs. ≥3), NLR (<2.5 or ≥2.5), and LDH (≤ ULN vs. >ULN) (*P* < 0.05; Supplementary Table 3). The discriminative performance (*c*-statistic) of the PFS model coefficients were 0.60.

The backwards deletion process resulted in an OS pre-treatment prognostic model which included LDH (≤ ULN vs. >ULN), metastatic sites count (<3 vs. ≥3), and ECOG performance status (0 vs. ≥1) (*P* < 0.001; Supplementary Table 4). The discriminative performance (*c*-statistic) of the OS model coefficients were 0.65.

**Figure 1:**
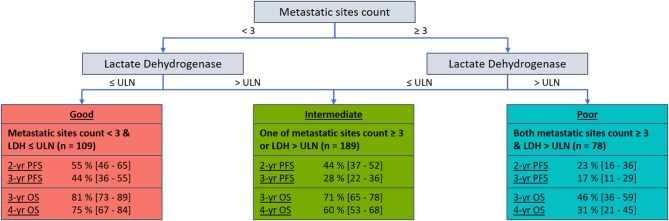
Decision tree with 2 and 3-year PFS, plus 3 and 4-year OS probabilities [95% CI] by pre-treatment prognostic groups for HER2-positive ABC patients who received first-line pertuzumab, trastuzumab, and docetaxel within CLEOPATRA.

**Figure 2:**
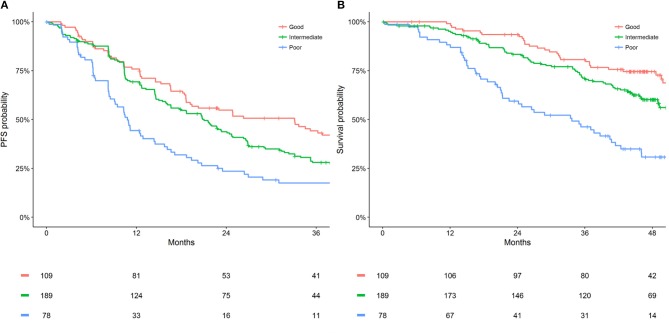
Kaplan Meier estimates of **(A)** PFS and **(B)** OS by pre-treatment prognostic groups for HER2-positive ABC patients who received first-line pertuzumab, trastuzumab and docetaxel within CLEOPATRA.

Metastatic sites count (<3 vs. ≥ 3) and LDH (≤ ULN vs. >ULN) were identified as common prognostic factors for PFS and OS. Subsequently, categorizing participants as one of three prognostic groups was assessed (good = 0 factors [neither metastatic sites count ≥ 3 or LDH > ULN]; intermediate = 1 factor [one of metastatic sites count ≥ 3 or LDH > ULN]; poor = 2 factors [both metastatic sites count ≥ 3 or LDH > ULN]). The intermediate and poor prognostic groups were significantly associated with worse PFS (HR [95% CI]: intermediate = 1.44 [1.07–1.95]; poor = 2.31 [1.63–3.27]; *P* < 0.001) and OS (HR [95% CI]: intermediate = 1.58 [1.04–2.40]; poor = 3.50 [2.22–5.51]; *P* < 0.001) compared to the good prognostic group. The discriminative performance (c-statistic) of these pre-treatment prognostic groups were 0.58 for PFS and 0.62 for OS, maintaining adequate performance with significant model simplification. Figure 1 presents the 2 and 3-year PFS, plus 3, and 4-year OS probabilities for the pre-treatment prognostic groups. Figure 2 presents the Kaplan-Meier plots of PFS and OS for the pre-treatment prognostic groups.

## Discussion

A pre-treatment prognostic tool for predicting long-term PFS and OS and probability in HER2-positive ABC patients commencing first-line pertuzumab, trastuzumab, and docetaxel was developed based on large (*n* = 408) and high-quality data, and to the best of the authors knowledge is the first study to present a prognostic tool for this patient group. The tool was able to clearly distinguish pre-treatment prognostic groups with significant differences in PFS and OS.

Metastatic sites count (<3 vs. ≥3) and LDH (≤ ULN vs. >ULN) were identified as common prognostic factors for PFS and OS. These predictors are in concordance with previous literature, where high pre-treatment metastatic burden (10, 11) and LDH (13–15) have been associated with poor outcomes in ABC. NLR (<2.5 or ≥2.5) was also identified as a significant prognostic factor of PFS, and ECOG performance status (0 vs. ≥1) for OS. The disparities between optimal PFS and OS prediction may be influenced by chance, disparity in PFS and OS correlation, and variable subsequent therapies between patients following study completion.

This study was conducted with a focus on developing a practical pre-treatment prognostic tool for clinical use. This included using routinely available clinical data, a single model for PFS and OS, and avoiding complex numerical derivations. While more complex models or development techniques [e.g., least absolute shrinkage and selection operator (LASSO) analysis] may result in greater prediction performance, simplicity of model use was prioritized to facilitate clinical use. The initial more complex PFS and OS prognostic models are reported in Supplementary Tables 3, 4 should optimal prediction performance be preferred.

The pre-treatment prognostic tool developed allows the simultaneous interpretation of PFS and OS prognostic risk for HER2-positive ABC patients who are considering first-line pertuzumab, trastuzumab, and docetaxel. The presented PFS and OS probabilities are only applicable to HER2-positive ABC patients who have not received previous treatment for their advanced disease (adjuvant or neoadjuvant therapies, with or without anti-HER2 agents, were permitted within CLEOPATRA) (2–4, 16). Notably this study observed a difference of 27% (44 vs. 17) in 3-year PFS probability between the good and poor prognostic groups for patients who received pertuzumab, trastuzumab, and docetaxel from CLEOPATRA. Furthermore, a difference of 44% (75 vs. 31) was observed in 4-year OS probability between the good and poor prognostic groups. Such discrimination between prognostic groups indicates a potential for the developed tool to provide more realistic expectations of long-term disease control and survival to HER2-positive ABC patients considering first-line pertuzumab, trastuzumab, and docetaxel.

Randomized clinical trials are the backbone of evidence-based medicine, and the data collected within are high-quality, stringently regulated, and herein contained PFS which is often not collected in real-world data yet important to patients. However, the inclusion criteria of trials can limit generalizability (17). Further, the study is a *post-hoc* analysis, rather than pre-planned. The discriminative performance of the prognostic groups for OS was consistent with a moderately well-performing model (*c* > 0.6) (18). Ideally the discriminative performance of the prognostic groups for PFS would have a concordance statistic >0.6, albeit a significant association between prognostic groups and PFS was identified (*P* < 0.001). This study presents a model developed using data which underpins the use of pertuzumab, trastuzumab, and docetaxel as a first-line therapy in the ABC population. The model may be used to better understand likely survival outcomes from therapy than is currently possible. While, the arrival of large electronic health record platforms will present important opportunities to externally validate and recalibrate the presented model, which may increase generalizability and performance.

In conclusion, a pre-treatment prognostic tool for PFS and OS in HER2-positive ABC patients initiating first-line pertuzumab, trastuzumab, and docetaxel was developed. The selected variables were in concordance with the previous literature and are routinely available in the clinic. The pre-treatment prognostic groups displayed significant difference in PFS and OS outcomes. There is the potential for the developed pre-treatment risk prediction tool to provide HER2-positive ABC patients with more realistic expectations of long-term disease control and survival when considering first-line pertuzumab, trastuzumab, and docetaxel therapy.

## Data Availability

Individual-participant data utilized in this study is available for request to access at clinicalstudydatarequest.com.

## Ethics Statement

Secondary analysis of anonymized participant-level trial data was approved by the Southern Adelaide Clinical Human Research Ethics Committee.

## Author Contributions

All authors were involved in analysis and manuscript preparation for this project.

### Conflict of Interest Statement

MS and AR report investigator-initiated project grants from Pfizer, outside the scope of the submitted work. The remaining authors declare that the research was conducted in the absence of any commercial or financial relationships that could be construed as a potential conflict of interest.
