# Supplementary material for: Predictors of Long-Term Disease Control and Survival for HER2-Positive Advanced Breast Cancer Patients Treated With Pertuzumab, Trastuzumab, and Docetaxel
Source: Front Oncol. 2019 Aug 22;9:789. doi: 10.3389/fonc.2019.00789 (PMC6713878; doi:10.3389/fonc.2019.00789)
Supplement: Supplementary file 1 [file Table_1.DOCX]

# Supplementary

Supplementary Table 1: Summary of patient characteristics

|  | **Total** |
| --- | --- |
|  | **No. 408** |
| Actual treatment given | |
| Pertuzumab + Trastuzumab + Docetaxel | 408 (100%) |
| Female | 408 (100%) |
| Age (years) |  |
| ≤ 60 | 307 (75%) |
| > 60 | 101 (25%) |
| ECOG performance status | |
| 0 | 278 (68%) |
| ≥ 1 | 130 (32%) |
| Race | |
| White | 250 (61%) |
| Non-White | 157 (38%) |
| Missing | 1 (0%) |
| Estrogen and Progesterone Receptor Status | |
| ER and PR Positive | 106 (26%) |
| ER or PR Negative | 298 (73%) |
| Missing | 4 (1%) |
| Months from initial diagnosis | |
| < 6 | 168 (41%) |
| ≥ 6 | 217 (53%) |
| Missing | 23 (6%) |
| Any prior trastuzumab all settings | 48 (12%) |
| Any prior taxane all settings | 93 (23%) |
| Visceral disease site at baseline | 321 (79%) |
| Count of metastatis sites | |
| < 3 | 168 (41%) |
| ≥ 3 | 240 (59%) |
| Lactate dehydrogenase | |
| ≤ ULN | 254 (62%) |
| > ULN | 122 (30%) |
| Missing | 32 (8%) |
| Neutrophil to lymphocyte ratio | |
| < 2.5 | 204 (50%) |
| ≥ 2.5 | 200 (49%) |
| Missing | 4 (1%) |
| Data are number of patients (%). | |

Supplementary Table 2: Univariate cox proportional hazard analysis of potential predictors of OS and PFS for HER2-positive advanced breast cancer patients treated with first-line pertuzumab, trastuzumab and docetaxel from CLEOPATRA

|  |  | **PFS** |  | **OS** |  |
| --- | --- | --- | --- | --- | --- |
| **Variable** | **N** | **HR [95%CI]** | **P** | **HR [95%CI]** | **P** |
| **Age (years)** |  |  | 0.12 |  | 0.941 |
| ≤ 60 | 307 |  |  |  |  |
| > 60 | 101 | 0.80 [0.60-1.07] |  | 0.99 [0.69-1.41] |  |
| **ECOG performance status** | |  | 0.095 |  | 0.001 |
| 0 | 278 |  |  |  |  |
| ≥ 1 | 130 | 1.24 [0.97-1.59] |  | 1.69 [1.24-2.30] |  |
| **Race** | |  | 0.116 |  | 0.922 |
| Non-White | 157 |  |  |  |  |
| White | 250 | 1.21 [0.95-1.54] |  | 0.98 [0.72-1.34] |  |
| **ER and PR Status** | |  | 0.678 |  | 0.020 |
| ER and PR Positive | 106 |  |  |  |  |
| ER or PR Negative | 298 | 1.06 [0.81-1.38] |  | 1.55 [1.05-2.28] |  |
| **Visceral disease site at baseline** |  |  | 0.363 |  | 0.124 |
| No | 87 |  |  |  |  |
| Yes | 321 | 1.14 [0.86-1.52] |  | 1.35 [0.91-2.02] |  |
| **Months from initial diagnosis** | |  | 0.307 |  | 0.399 |
| < 6 | 168 |  |  |  |  |
| ≥ 6 | 217 | 0.88 [0.70-1.12] |  | 1.15 [0.83-1.58] |  |
| **Any prior trastuzumab all settings** |  |  | 0.049 |  | 0.646 |
| No | 360 |  |  |  |  |
| Yes | 48 | 1.44 [1.02-2.03] |  | 1.11 [0.71-1.76] |  |
| **Any prior taxane all settings** |  |  | 0.754 |  | 0.995 |
| No | 315 |  |  |  |  |
| Yes | 93 | 1.04 [0.79-1.37] |  | 1.00 [0.70-1.42] |  |
| **Metastatic sites count** | |  | <0.001 |  | <0.001 |
| < 3 | 168 |  |  |  |  |
| ≥ 3 | 240 | 1.64 [1.29-2.08] |  | 1.81 [1.31-2.50] |  |
| **Lactate dehydrogenase** | |  | 0.015 |  | <0.001 |
| ≤ ULN | 254 |  |  |  |  |
| > ULN | 122 | 1.37 [1.07-1.77] |  | 1.97 [1.43-2.71] |  |
| **Neutrophil to lymphocyte ratio** | |  | 0.001 |  | 0.179 |
| < 2.5 | 204 |  |  |  |  |
| ≥ 2.5 | 200 | 1.48 [1.17-1.87] |  | 1.23 [0.91-1.67] |  |

Supplementary Table 3: Coefficients for the multivariable Cox proportional hazard model specific for prediction of PFS in HER2-positive advanced breast cancer patients treated with first-line pertuzumab, trastuzumab and docetaxel

| Variable | Coefficient [95% CI] |
| --- | --- |
| Metastatic sites count ≥ 3 | 0.493 [0.240-0.747] |
| NLR ≥ 2.5 | 0.347 [0.103-0.591] |
| LDH > ULN | 0.298 [0.045-0.551] |

Supplementary Table 4: Coefficients for the multivariable Cox proportional hazard model specific for prediction of OS in HER2-positive advanced breast cancer patients treated with first-line pertuzumab, trastuzumab and docetaxel

| Variable | Coefficient [95% CI] |
| --- | --- |
| LDH > ULN | 0.615 [0.292-0.938] |
| Metastatic sites count ≥ 3 | 0.600 [0.261-0.939] |
| ECOG performance status ≥ 1 | 0.502 [0.178-0.826] |
